# Malnutrition in rectal cancer patients receiving preoperative chemoradiotherapy is common and associated with treatment tolerability and anastomotic leakage

**DOI:** 10.1007/s00384-016-2507-8

**Published:** 2016-02-18

**Authors:** Tomoki Yamano, Mie Yoshimura, Masayoshi Kobayashi, Naohito Beppu, Michiko Hamanaka, Akihito Babaya, Kiyoshi Tsukamoto, Masafumi Noda, Nagahide Matsubara, Naohiro Tomita

**Affiliations:** Division of Lower GI Surgery, Department of Surgery, Hyogo College of Medicine, 1-1 Mukogawa-cho, Nishinomiya, Hyogo 663-8501 Japan

**Keywords:** Malnutrition, Rectal cancer, Chemoradiotherapy, Anastomotic leakage

## Abstract

**Purpose:**

This study assessed the incidence of malnutrition caused by preoperative chemoradiotherapy (CRT) in rectal cancer patients, which is seemingly underestimated; however, malnutrition affects treatment tolerability, postoperative complications, including anastomotic leakage (AL), and oncological outcomes.

**Methods:**

Between January 2008 and December 2014, 54 consecutive patients with T3–4, N0–2, M0–1 resectable rectal cancer received CRT comprising 45 Gy radiotherapy and S-1 alone or with irinotecan for 5 weeks and then underwent curative surgery with diverting or permanent stomas 6–8 weeks after CRT. We assessed malnutrition after completion of CRT (5–6 weeks after CRT start date) and at surgery (11–14 weeks after CRT start date), defining weight loss as ≥5 % of pre-CRT weight; this definition differs from commonly used criteria for adverse events. We evaluated the incidence of malnutrition associated with CRT and influence of malnutrition on treatment tolerability, AL, and disease-free survival (DFS). We also assessed the influence of CRT on the rate of postoperative complications by comparing the study group with 61 patients who had undergone excision with diverting or permanent stomas alone.

**Results:**

Malnutrition was observed in 51 % of patients after CRT and in 29 % at surgery. Malnutrition after CRT was associated with treatment tolerability, and malnutrition at surgery was significantly associated with AL, which significantly influenced DFS in stage 1–3 patients.

**Conclusion:**

Malnutrition caused by CRT is common and is associated with treatment tolerability and AL. Nutritional assessment and support seem indispensable for the rectal cancer patients receiving CRT.

## Introduction

Colorectal cancer is one of the most common malignancies worldwide and is the second most common malignancy in Japan [1]. In Western countries, preoperative chemoradiotherapy (CRT) is the standard therapy for reducing the risk of local recurrence in rectal cancer [2]. Common adverse effects of CRT include hematological toxicities, diarrhea, appetite loss, and surgical site infection (SSI), including anastomotic leakage (AL). However, the relationship between CRT and SSI remains unclear [3–5].

Malnutrition has been recognized as a risk factor for postoperative complications, especially infections, including SSI and AL [6]. Nutritional status affects both treatment tolerability and survival in gastrointestinal cancer patients; however, the incidence of malnutrition is underestimated [7, 8]. The American Society for Parenteral and Enteral Nutrition guidelines do not recommend routine support therapy during anticancer treatment, and European Society for Clinical Nutrition and Metabolism (ESPEN) guidelines recommend nutritional support only for patients with malnutrition [9, 10]. The usefulness of nutritional intervention in cancer patients treated with radiotherapy (RT) remains controversial [11–14]. In patients with head and neck or esophageal cancer, CRT has been found to worsen nutritional status; the value of nutritional intervention in these cases is currently being assessed [15].

In rectal cancer patients, malnutrition caused by CRT is not recognized as a common adverse effect requiring nutritional support, although CRT is usually more toxic than RT alone. This is likely because grade 3 weight loss as defined by the Common Terminology Criteria for Adverse Events (CTCAE) version 4.0 is weight loss of 20 %, which is unlikely to occur in only 1 month of CRT treatment [16]. The malnutrition screening tool (MUST) and nutrition risk screening (NRS) adopt thresholds of 5 % or more weight loss in 3 months as indicators of malnutrition [17, 18].

Therefore, we evaluated the incidence of malnutrition by CRT at two different time points: after completion of CRT and at surgery, by criteria other than the CTCAE criteria (weight loss; threshold of 5 % or more). Other nutritional indicators, body mass index (BMI), serum albumin concentrations, and NRS were also assessed. The associations of malnutrition with treatment tolerability, SSI including AL, and disease-free survival (DFS) were evaluated.

We also assessed the influence of CRT on postoperative complications by comparing the study group with 61 rectal cancer patients who received curative surgery with permanent or diverting stomas without CRT during the same time period.

## Patients and methods

### Patients

Between June 2009 and December 2014, consecutive patients who were diagnosed clinically with T3 or T4, N0–2, M0–1a (resectable liver metastasis) rectal cancer were recommended to undergo CRT before surgery. Fifty-four patients consented to CRT comprising 45 Gy (25 fractions of 1.8 Gy on days 1–5, 8–12, 15–19, 22–26, and 29–33) in conjunction with S-1 (80 mg/m^2^ on days 1–5, 8–12, 22–26, and 29–33) and irinotecan (40–90 mg/m^2^ on days 1, 8, 22, and 29). Six to 8 weeks after the completion of CRT, total mesorectal excision with diverting stoma or permanent colostomy was performed on all patients. This protocol, which is based on that described by Sato et al., is the same procedure as that used in the S-1 combined preoperative neoadjuvant Multimodality therapy with Radiation and Irinotecan for locally advanced rectal cancer (SAMRAI)-1 trial and SAMRAI-2 trial (UMIN000001639 and UMIN000011115, respectively) [19]. To assess the influence of CRT on postoperative complications, the study patients’ data were compared with those of 61 patients who underwent the Miles operation, Hartmann operation, or low anterior resection/intersphincter resection with diverting stomas according to the individual surgeon’s judgments to minimize complications related to AL during the same time period. These 61 patients consisted of those who were not eligible for CRT or those who refused CRT. They were considered as a high-risk group for postoperative complications. All patients were histologically confirmed to have rectal adenocarcinoma before commencing treatment. The diagnoses were based on clinical examination and the results of pelvic magnetic resonance imaging and chest/abdominal computed tomography (CT) data. Data collected included clinical and pathological features, adverse effects during CRT, perioperative complications, and prognosis. Pathological features were classified according to the seventh edition of the American Joint Committee on Cancer/tumor node metastasis system. Patients with complete preoperative responses were classified as stage 0. Among 54 patients, five patients were excluded from analysis because they were enrolled in the SAMRAI-2 trial, in which nutritional assessment was one of the secondary endpoints. One patient received S-1 alone because of general fatigue that would have been exacerbated by CRT that included irinotecan. Informed consent was obtained from all included patients and the Medical Ethics Committee of the hospital approved this study. Follow-up information on postoperative recurrence and survival was obtained by mail for patients who were followed up elsewhere. DFS was defined as time (in months) from surgery to the first evidence of any further malignancy, death, or last follow-up with no events (for censored patients). The mean and median durations of follow-up were 37.8 and 36 months, respectively.

### Nutritional assessment

Serum albumin concentrations were measured using routine methods. Height was routinely measured in all patients, and weight was measured using a digital scale. Body weight and serum albumin concentrations were measured at three different time points (before CRT, after CRT, and at surgery) for CRT(+) patients and at surgery for CRT(−) patients. The time point of “on consultation” means before CRT in CRT(+) patients or at surgery in CRT(−) patients. BMI was calculated as weight/height^2^ (kg/m^2^). Patients were classified according to the World Health Organization criteria as follows: underweight (BMI < 18.5), normal weight (18.5 ≤ BMI < 25), overweight (25 ≤ BMI < 30), and obese (BMI ≥ 30). NRS before CRT, after CRT, and at surgery was calculated according to the ESPEN guidelines [10]. The duration of CRT was approximately 5 to 6 weeks, and the period between the start of CRT and surgery approximately 3 months. Malnutrition was considered as weight loss of ≥5 % after CRT or at surgery, compared with the patient’s weight before CRT.

### Dose intensity of chemotherapy and event monitoring

Dose intensity was calculated as the ratio of the total dosage administered to patients to that scheduled during CRT, which was 1600 mg/m^2^ for S-1 and 240 mg/m^2^ for irinotecan. Receipt of scheduled doses of both S-1 and irinotecan (100 % or more) was categorized as “complete” dose intensity.

Adverse effects of CRT such as hematological and non-hematological toxicities were recorded and graded according to the CTCAE version 4.0. All patients were followed up for more than 30 days after surgery to assess morbidity. The diagnosis of SSI was based on the definition in Japan nosocomial infections surveillance guidelines [20]. AL was confirmed using CT or contrast enema upon presentation with clinical symptoms, which included abdominal pain, fever, and leukocytosis. Some asymptomatic patients were found to have AL by routine contrast enema 5–7 days after surgery.

### Statistical analysis

Statistical analyses were performed using JMP version 11 (SAS Japan Inc., Tokyo, Japan). The influence of CRT on clinical characteristics, including BMI, serum albumin, NRS, SSI, and AL, was assessed using a two-sided *t* test or the *χ* ^2^ test. The relationships between malnutrition after CRT or at surgery and side effects, dose intensity, and efficacy of CRT were assessed using the *χ* ^2^ test. Nutritional indicators and other factors that have previously been reported to have an association with AL were analyzed in the 86 patients who received surgery with anastomosis using univariate and multivariate analyses with a logistic regression model [3–5]. DFS was also assessed in 76 patients who received surgery with anastomosis and were classified as having stage 1–3 disease, by a log-rank test. All differences with *P* value of <0.05 were considered statistically significant.

## Results

### General patient characteristics

Patient characteristics are summarized in Table 1. There were no significant differences in sex, age, presence of diabetes mellitus, distance from the anal verge, blood loss, operation time, and incidence of SSI between CRT(+) patients and CRT(−) patients. CRT(+) patients were more likely to take steroids and have a smoking habit. CRT(−) patients were more likely to be classified as category 3 on the American Society of Anesthesiologists Physical Status criteria, but were less likely to receive anastomosis with surgery. The proportion of stages also differed between CRT(+) and CRT(−) patients; there were more stage 0 or 2 patients in the CRT(+) than the CRT(−) group, perhaps because CRT resulted in downstaging of some patients. We consider that these differences between CRT(+) and CRT(−) patients were inevitable, because this study retrospectively, but not prospectively, enrolled CRT(−) patients who had stomas during the study period. Because CRT(−) patients had high American Society of Anesthesiologists scores and were therefore considered at high risk of postoperative complications, diverting stomas were constructed to reduce complications related to anastomotic leakage. Therefore, we could not conclude from our data that CRT increases the incidence of anastomotic leakage.

**Table 1:** Characteristics of the patients (*N* = 110)

Patient characteristics	CRT(+)	CRT(−)	*P* CRT(+) vs.
	(*n* = 49)	(*n* = 61)	CRT(−)
Gender (M/F)	36/13	37/24	0.22
Age	63.1	64.7	0.51
Diabetes mellitus	7	8	1.0
Steroid	4	0	0.04
Smoking	14	7	0.029
ASAPS (1/2/3)	4/37/8	14/32/15	0.033
BMI			
On consultation	23.1	22.4	0.35
After CRT	21.7 (*P* < 0.0001)	–	
At surgery	22.4 (*P* = 0.0006)	22.4	0.98
Albumin(g/dL)			
On consultation	4.1	4.2	0.17
After CRT	3.3 (*P* < 0.0001)	–	–
At surgery	4.0 (*P* = 0.06)	4.2	0.0032
NRS ≥ 3			
On consultation	11	15	0.79
After CRT	29	–	–
At surgery	20	15	0.07
AV (mm)	43.1	41.3	0.61
Blood loss (mL)	614	666	0.66
Operation time (min)	279	275	0.80
Miles/Hartman/LAR/ISR	5/0/20/24	15/4/14/28	0.027
T (0 or CR/1/2/3/4)	6/3/10/24/6	2/13/12/28/6	0.11
N (0/1/2)	38/8/3	36/16/9	0.11
Stage (0 or CR/I/II/III/IV)	6/9/22/8/4	2/19/14/18/8	0.022
SSI	17 (34.7 %)	12 (19.7 %)	0.076
Anastomotic leakage	12 (27.3 %)	4 (9.5 %)	0.03
Adjuvant therapy in stages 0–1	5 (33.3 %)	0 (0 %)	0.004
Adjuvant therapy in stage 2	9 (40.9 %)	3 (21.4 %)	0.22
Adjuvant therapy in stage 3	6 (75 %)	12 (66.7 %)	0.67
Events in stages 0–3	13 (28.9 %)	13 (24.5 %)	0.79
Recurrence in stages 0–3	12 (26.7 %)	8 (15.1 %)	0.16

*CRT* chemoradiotherapy, *ASAPS* American Society of Anesthesiologists Physical Status, *BMI* body mass index, *NRS* nutritional risk screening, *AV* distance from the anal verge, *LAR* low anterior resection, *ISR* intersphincter resection, *SSI* surgical site infection

A complete response was achieved in six of the 49 patients (12 %) who received CRT. Four CRT(+) patients had stage 4 disease: two received CRT because they had resectable liver metastases, and the remaining two were diagnosed as having unresectable distant lymph node metastases or resectable liver metastases after CRT. The three with liver metastases subsequently underwent liver resection.

### Changes in nutritional status

There were no significant differences in BMI, albumin concentrations, and the proportion of patients with NRS ≥3 between CRT(+) and CRT(−) patients on consultation. However, BMI and albumin concentrations were significantly lower and the proportion of patients with NRS ≥3 was greater in the CRT(+) group. Although albumin concentrations at surgery had recovered to those before CRT, BMI at surgery was still significantly lower than that before CRT (Table 1). There were still more patients with NRS ≥3 at surgery than there had been before CRT; however, the proportion of patients with NRS ≥3 did not differ significantly between CRT(+) and CRT(−) patients.

Because the usefulness of routine nutritional intervention for patients receiving preoperative CRT has not yet been shown, routine nutritional interventions were not implemented except in one patient who requested oral supplementation. Four patients with grade 3 adverse effects received peripheral intravenous nutrition to relieve their symptoms.

**Fig. 1:**
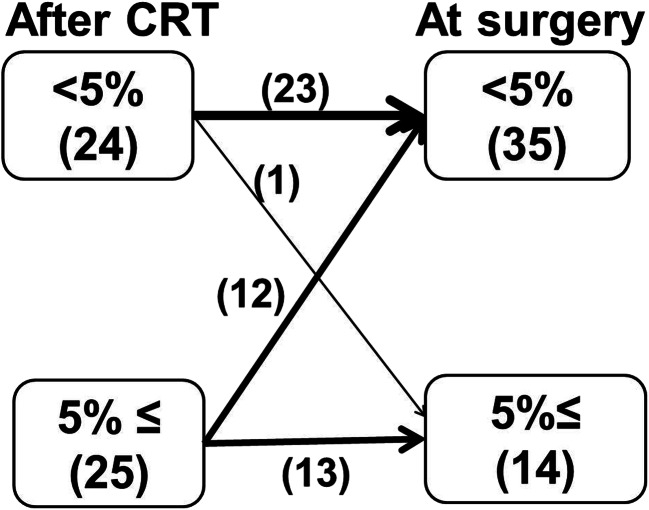
Changes in weight during the period between completion of chemoradiotherapy (*After CRT*) and surgery (*At surgery*)

Figure 1 shows the changes in weight loss after CRT and at surgery. Weight loss of ≥5 % (malnutrition) was observed in 51 % of patients (25/49) after CRT. Malnutrition at surgery was noted in 29 % of patients (14/49). Most of them (13/49) sustained malnutrition since after CRT.

### Relationship between malnutrition and adverse effects/efficacy

Loss of appetite was significantly associated with malnutrition after CRT (*P* < 0.0001) and at surgery (*P* = 0.011) (Table 2). However, diarrhea was significantly associated with malnutrition after CRT (*P* < 0.05), but not with malnutrition at surgery. Hematological toxicities or histological grade (CRT efficacy) was not significantly associated with malnutrition after CRT or at surgery (Table 2). Other non-hematological grade 3 and 4 toxicities included peripheral artery ischemia (one patient), pneumonia (one patient), and gastric perforation (one patient).

**Table 2:** Relationship between malnutrition and side effects/efficacy (*N* = 49)

Factors	Grade	Malnutrition after CRT			Malnutrition at surgery		
	Yes/no	(−)	(+)	*P*	(−)	(+)	*P*
Appetite loss	0	21	0	<0.0001	20	1	0.011
	1	1	12		6	7	
	2	2	9		7	4	
	3	0	4		2	2	
Diarrhea	0	14	11	0.033	16	9	0.18
	1	3	3		5	1	
	2	6	2		8	0	
	3	1	9		6	4	
Hematological toxicities	0	3	4	0.37	5	2	0.26
	1	8	5		11	2	
	2	8	13		13	8	
	3	5	2		6	1	
	4	0	1		0	1	
Complete dose intensity	Yes	19	12	0.022	25	6	0.06
	No	5	13		10	8	
Histological response	1	12	14	0.51	17	9	0.47
	2	10	7		14	3	
	3	2	4		4	2	

*CRT* chemoradiotherapy

In total, 63 % (31/49) of patients received the scheduled dose of chemotherapy. Malnutrition after CRT was significantly associated with completion of chemotherapy (*P* = 0.022): 79 % (19/24) of patients without malnutrition received the complete dose of chemotherapy, whereas 48 % (12/25) of patients with malnutrition received the complete dose of chemotherapy (Table 2). These results indicated that severe appetite loss and diarrhea induced by CRT resulted in malnutrition and decreased dose intensity.

### Incidence and treatment of anastomotic leakage

Eighty-six patients who had undergone low anterior or intersphincteric resection (44 patients who did receive CRT and 42 patients who did not) were assessed for anastomotic leakage (Table 1). The incidence of AL was significantly greater (27.3 %) in CRT(+) than in CRT(−) patients (9.5 %). Six of 12 patients with AL in the CRT(+) group required no further treatment. The symptoms of three of the other six resolved with antibiotics; the remaining three underwent surgery for peritonitis. Of four patients with AL in the CRT(−) group, two required no further treatment and the other two received antibiotics.

### Risk factors associated with anastomotic leakage

Risk factors associated with AL were further analyzed in a subgroup of 86 patients with anastomoses consisting of 44 CRT(+) and 42 CRT(−) patients. In univariate analysis, CRT, serum albumin concentration at consultation <3.5 g/dL, and malnutrition at surgery were significantly associated with AL (Table 3). In subsequent multivariate analysis using the factors with less than 0.1 of the *P* value, malnutrition at surgery was identified as an independent risk factor for AL in patients who received CRT (Table 3). However, CRT and albumin concentration at consultation <3.5 g/dL were not significant independent risk factors for AL when all patients were analyzed.

**Table 3:** Characteristics of the patients with or without anastomosis and univariate/multivariate logistic regression analysis of risk factors associated with anastomotic leakage (*N* = 86)

Factors	AL(+)	AL(−)	Univariate analysis		Multivariate analysis			
					All patients		CRT(+) patients	
	(*N* = 16)	(*N* = 70)	Odds ratio	*P*	Odds ratio	*P*	Odds ratio	*P*
Age ≥75	3	10	1.4	0.65				
Sex (male)	10	53	0.53	0.29				
Diabetes mellitus	4	9	2.3	0.22				
Steroid	2	1	9.9	0.07	3.8	0.31	3.9	0.38
Smoking	2	18	0.41	0.27				
CRT (*N* = 44)	12	32	3.6	0.03	3.3	0.056		
Albumin on consultation <3.5 g/dL	3	2	7.8	0.03	1.1	0.95	1.3	0.84
Albumin at surgery <3.5 g/dL	1	4	1.1	0.93				
Malnutrition after CRT	7	14	1.8	0.39				
Malnutrition at surgery	6	5	5.4	0.024			6.3	0.02
ASAPS = 3	5	9	3.1	0.08	2.8	0.17	4.6	0.14
BMI underweight on consultation	1	7	0.6	0.8				
BMI underweight at surgery	2	7	1.3	0.49				
NRS ≥ 3 on consultation	3	13	1.0	0.99				
NRS ≥ 3 after CRT	8	17	1.8	0.42				
NRS ≥ 3 at surgery	7	16	2.6	0.1				
Blood loss >500 mL	9	28	1.9	0.24				
Operation time >5 h	8	21	2.3	0.14				
AV < 40 mm (*N* = 29)	5	24	0.9	0.82				
Stage ≥ 2 (*N* = 52)	12	40	2.3	0.18				

*AL* anastomotic leakage, *CRT* chemoradiotherapy, *BMI* body mass index, *NRS* nutritional risk screening, *AV* distance from the anal verge, *ASAPS* American Society of Anesthesiologists Physical Status

### Oncological outcomes

**Fig. 2:**
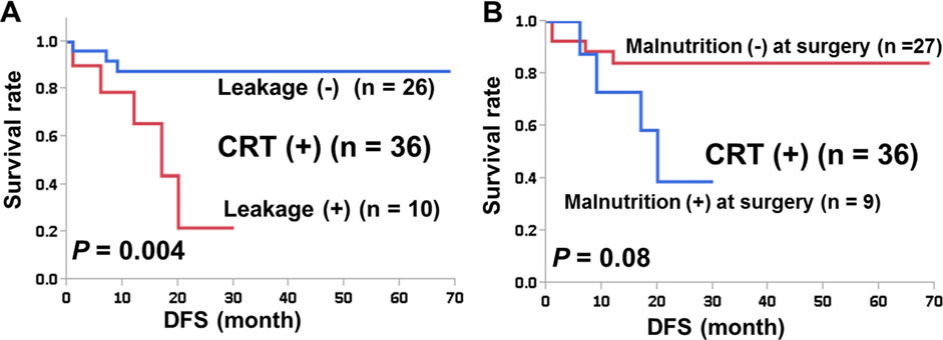
Survival curves by the Kaplan-Meier method for patients with stage 1–3 rectal cancer who had anastomoses constructed. **a** Patients who underwent both CRT and surgery (CRT(+)) according to the presence (+) or absence (−) of anastomotic leakage. **b** Patients who underwent both CRT and surgery (CRT(+)) according to the presence (+) or absence (−) of malnutrition at surgery. *DFS* disease-free survival

Figure 2 shows that AL was a significant prognostic factor in CRT(+) (*n* = 36, *P* = 0.004). DFS at 2 years was 22 % in CRT(+) patients with AL and 89 % in CRT(+) patients without AL. When stage 0 patients were included, DFS at 2 years was 44 % in CRT(+) patients with AL. Malnutrition at surgery was not a significant prognostic factor for AL (*P* = 0.08).

Five of ten patients with AL developed recurrence (one stage 1, three stage 2, and one stage 3). Two stage 2 and one stage 3 patients received adjuvant chemotherapy. Five patients without recurrences received no adjuvant chemotherapy.

## Discussion

In this study, we evaluated malnutrition at three different time points (on presentation, after CRT, and before surgery) using criteria other than those of the CTCAE. We defined malnutrition as 5 % weight loss compared with before CRT and found that malnutrition in patients with rectal cancer receiving CRT is common and that sustained malnutrition is associated with AL. BMI and serum albumin concentrations were significantly reduced following CRT, although serum albumin concentrations recovered during the following 6–8 weeks. NRS also deteriorated with CRT; however, malnutrition defined as NRS ≥3 was not associated with AL. These results demonstrate that CRT for rectal cancer affects nutritional status adversely and some patients still have malnutrition 6–8 weeks after CRT.

Malnutrition has been recognized as a risk factor for postoperative complications and oncological outcomes [6–8]. However, in rectal cancer patients, malnutrition associated with CRT has not been recognized as a common adverse effect [19, 21]. This may be partly because adverse events associated with CRT are usually evaluated by CTCAE, in which severe (grade 3) weight loss is defined as 20 % weight loss during CRT or the necessity for total parenteral nutrition or tube feeding. It seems close to impossible that grade 3 body weight loss could occur during the 5 weeks of our CRT protocol.

In our study, the incidence of AL in CRT(+) patients was significantly higher (27.3 %) than in CRT(−) patients (9.5 %). Although this percentage is higher than in previous studies, this retrospective case series included patients with factors that made them unsuitable for admission to a prospective clinical trial (four patients were receiving steroids and two patients had a history of other cancer within the previous 5 years).

The mean distance from the anal verge (about 4 cm) was smaller than in previous studies; more than half the patients underwent intersphincter resection [3–5]. Tumor location and difficulty of anastomotic procedure are reportedly associated with incidence of AL [3]. Therefore, we consider these factors to account for our high incidence of AL. Schiffmann et al. reported the same incidence of AL in a retrospective study [22]. Based on our data, we recommend defunctioning stomas after CRT in patients who have undergone low rectal cancer surgery [23].

Our sample was small and the data were assessed retrospectively in a single institute. The incidence of malnutrition and adverse events in patients subjected to the protocol used in this study seems relatively high compared with previous reports. Therefore, in October 2013, we started a phase II trial using irinotecan with S-1 and RT for rectal cancer (SAMRAI-2 trial, UMIN000011115) based on the findings of the present study. In this new trial, we will assess the relationships between nutritional indicators and adverse effects, postoperative complications, and DFS.

Nutritional support before gastrointestinal surgery is recommended for patients at severe nutritional risk but is controversial for patients without clinical evidence of malnutrition [9, 10, 24–26].

Our study will encourage evaluating the nutritional status of rectal cancer patients receiving CRT by criteria other than those of the CTCAE and providing nutritional support for patients with malnutrition to reduce the incidence of AL.
